# LncRNA POU3F3 promotes melanoma cell proliferation by downregulating lncRNA MEG3

**DOI:** 10.1007/s12672-021-00414-9

**Published:** 2021-07-09

**Authors:** Yingnan Liu, Yongqing Zhuang, Xiaokuan Fu, Chaofei Li

**Affiliations:** 1 Department of Hand and Microvascular Surgery, Shenzhen People’s Hospital, The Second Clinical Medical College, Jinan University, Shenzhen, 518000 Guangdong China; 2 Department of Hand and Microvascular Surgery, Shenzhen People’s Hospital, The First Affiliated Hospital,Southern University of Science and Technology, Shenzhen, 518000 Guangdong China; 3 Department of General Surgery, Ruijin Hospital Affiliated to Shanghai Jiao Tong University School of Medicine, 12th floor, Building 9, No. 197, Ruijin 2nd Road, Huangpu District, Shanghai, 200025 China

**Keywords:** Melanoma, LncRNA POU3F3, LncRNA MEG3, Proliferation

## Abstract

**Background:**

LncRNA POU3F3 (POU3F3) is overexpressed and plays oncogenic roles in esophageal squamous-cell carcinomas. LncRNA MEG3 (MEG3) has been characterized as a tumor suppressive lncRNA in different types of cancer. Our preliminary deep sequencing analysis revealed the inverse correlation between POU3F3 and MEG2 across melanoma tissues, indicating the interaction between them in melanoma. Therefore, this study was performed to investigate the crosstalk between POU3F3 and MEG3 in melanoma.

**Methods:**

Tumor and adjacent healthy tissues collected from 60 melanoma patients were subjected to RNA extractions and RT-qPCRs to analyze the differential expression of POU3F3 and MEG2 in melanoma. In melanoma cells, POU3F3 and MEG2 were overexpressed to study the interactions between them. CCK-8 assays were performed to analyze the roles of POU3F3 and MEG2 in regulating melanoma cell proliferation.

**Results:**

We found that POU3F3 was upregulated, while lncRNA MEG3 was downregulated in melanoma. Expression levels of POU3F3 and MEG3 were inversely correlated across tumor tissues. In vitro experiments showed that POU3F3 overexpression decreased MEG3 expression in melanoma cells, while MEG3 overexpression failed to affect POU3F3. POU3F3 overexpression increased melanoma cell proliferation, while MEG3 overexpression decreased melanoma cell proliferation. In addition, rescue experiments showed that MEG3 overexpression attenuated the enhancing effects of POU3F3 overexpression.

**Conclusion:**

POU3F3 may promote melanoma cell proliferation by downregulating MEG3.

## Background

Long non-coding RNAs, or lncRNAs, are non-protein-coding RNAs with a length longer than 200 nucleotides [1]. It has been shown that the human genome contains more than 70,000 genes that transcribe lncRNAs, even more than protein-coding genes [2]. There is mounting evidence showing that lncRNAs have important roles in human diseases, especially in human cancers [3]. Cancer development and progression globally affect the expression of lncRNAs, and the differentially expressed lncRNAs may play roles as oncogenes or tumor suppressors to participate in cancer biology [4]. Investigation of the expression pattern and functionality of lncRNAs in cancer revealed that expression regulation of some key regulator lncRNAs might serve as a promising therapeutic target for cancer therapy [5].

Although melanoma is not a common malignancy in many countries, such as China, its incidence has increased more rapidly than most other cancers in the past several decades, making it possible to become a common malignancy in clinical practices in the near future [6, 7]. Therefore, investigating the molecular mechanism of melanoma pathogenesis is of great clinical value. Although lncRNAs are critical determinants of melanoma, their clinical application values are unclear due to their obscure biological functions [8–10]. LncRNA POU3F3 (POU3F3) was first characterized as a potential oncogene in esophageal squamous-cell carcinomas [11]. Following studies have shown that POU3F3 participate in many types of cancers, such as breast cancer [12], nasopharyngeal carcinoma [13] and prostate carcinoma [14] mainly by regulating cancer cell behaviors through regulating cancer-related genes, such as caspase 9, TGF-β1 and rho-associated protein kinase 1 [12–14]. LncRNA MEG3 (MEG3) has been characterized as a tumor suppressive lncRNA in different types of cancers, and MEG3 upregulation inhibits cancer development [15, 16]. In this study, we proved that POU3F3 promoted melanoma cell proliferation possibly by downregulating MEG3, a tumor suppressor in melanoma [17].

## Methods

### Research subjects

Our study included 60 melanoma patients who were admitted to Ruijin Hospital Affiliated to Shanghai Jiao Tong University School of Medicine from January 2015 to August 2018. All patients understood the aims of specimen collection and signed written informed consent. The inclusion criteria were (1) patients with melanoma and biopsies were confirmed by 3 experienced pathologists; and (2) patients who had not been treated by any treatments before admission. The exclusion criteria were (1) patients who were unwilling to donate biopsies; (2) patients complicated with other diseases; and (3) patients who were transferred from other hospitals or had been treated before admission. The 60 melanoma patients included 36 males and 24 females, and the mean age was 46.7 ± 8.0 years. According to the American Joint Committee on Cancer (AJCC) staging criteria, the 60 patients were classified into stage IA (n = 5), IB (n = 8), IIA (n = 8), IIB (n = 6), IIC (n = 5), IIIA (n = 9), IIIB (n = 5), IIIC (n = 4) and IV (n = 10), respectively. Preoperative chemotherapy or radiotherapy was not performed on the patients and all tissue samples were confirmed through histopathologic diagnosis by three independent pathologists. Tumor size was > 15 mm in 28 cases and ≤ 15 mm in 32 cases. Tumor thickness was > 10 mm in 34 cases and ≤ 10 mm in 26 cases. Ethical approval was obtained from the Ethics Committee of Ruijin Hospital Affiliated to Shanghai Jiao Tong University School of Medicine. The research was carried out in accordance with the World Medical Association Declaration of Helsinki 2013. Table 1 lists the clinical information of the enrolled patients.

**Table 1:** Clinical information of the 60 melanoma patients

Parameters	Cases
Age (years)	
> 45	27
≤ 45	33
Gender	
Male	36
Female	24
AJCC stages	
IA	5
IB	8
IIA	8
IIB	6
IIC	5
IIIA	9
IIIB	5
IIIC	4
IV	10
Tumor size (mm)	
> 15	28
≤ 15	32
Tumor thickness (mm)	
> 10	34
≤ 10	26
Extrascleral extension	
Yes	8
No	52

### Tissues, cell lines and cell transfection

Tumor and adjacent healthy (normal) tissues (about 0.03 g) within the area about 2 cm around tumors were collected from each participant. Healthy tissues and tumor tissues were confirmed by at least three pathologists. Tumor cell content in tumor was higher than 70% and tumor cell content in healthy tissues was below 1%. Tissues were stored in liquid nitrogen before RNA extraction.

Our study included two human melanoma cell lines, A375 and M21. These cells were obtained from ATCC (Manassas, VA, USA) and cultured in Eagle’s Minimum Essential Medium (Catalog No. 30-2003) containing 10% fetal bovine serum at 37 °C and in a humidified incubator with 5% CO_2_.

POU3F3 and MEG3 overexpression vectors were constructed by GeneCopoeia (Guangzhou, China). POU3F3 and MEG3 siRNAs were from RiboBio (Guangzhou, China). A375 and M21 cells were cultured to 70–80% confluence before cell transfections. All cell transfections were performed using Lipofectamine 3000 (Thermo Fisher Scientific) with 10 nM vectors or 10 nm siRNA in the transfection mixture. Cells were collected at 24 h after transfection to perform subsequent experiments. Lipofectamine 3000 only-treated cells were used as the control. Empty vector-transfection and NC siRNA-transfection were used as the negative controls.

### RT-qPCR

Tissue specimens were ground in liquid nitrogen, followed by the addition of RNAzol^®^ RT RNA Isolation Reagent (Sigma-Aldrich). In vitro cultured cells were directly mixed with RNAzol^®^ RT RNA Isolation Reagent to extract total RNA. Expression levels of POU3F3 and MEG3 were measured by RT-qPCR. Briefly, total RNA samples were reversely transcribed into cDNA using SuperScript IV Reverse Transcriptase (Thermo Fisher Scientific). Luna^®^ Universal One-Step RT-qPCR Kit (NEB) was used to prepare PCR mixtures with SYBR Green (NEB) as the fluorophore. With GAPDH as endogenous control, qPCRs were performed through the following conditions: 2 min at 95 °C, followed by 40 cycles of 15 s at 95 °C and 40 s at 55 °C. All data normalizations were performed according to the 2^−ΔΔCT^ method [18]. Primer sequences were: MEG3 forward 5′-CTGCCCATCTACACCTCAC-3′ and reverse 5′-CTCTCCGCCGTCTGCGCTA GGG-3′, POU3F3 forward 5′-TCATCCTTCAGRGRCCATCC-3′ and reverse 5′-ATC TCAGATTCCTGGGCTGG-3′, GAPDH forward 5′-ACCACAGTCCATGCCATCA-3′ and reverse 5′-CCACCACCCTGTTGCTGTA-3′.

### Measurement of cell proliferation ability

Cell Counting Kit-8 (CCK-8) assay was performed to measure cell proliferation ability using a kit provided by Sigma-Aldrich. Briefly, single cell suspensions (3 × 10^4^ cells/ml) were prepared using Eagle's Minimum Essential Medium (10% FBS). A 96-well plate was used to culture cells with 3 × 10^3^ cells (0.1 ml) in each well at 37 °C and 5% CO_2,_ and 10 µl CCK-8 was added at 24, 28, 72 and 96 h after the initiation of cell culture. Cell proliferation abilities were represented by OD values at 450 nm, which were measured using a microplate reader (Bio-Rad, USA).

### Measurement of cell migration and invasion ability

Corning^®^ HTS Transwell^®^ 96 well Transwell (8.0 μm, CLS3384, Sigma-Aldrich) was used to measure cell migration and invasion. Matrigel (356234, Millipore, USA) was used to coat the upper chamber before invasion assay. Briefly, single cell suspensions (3 × 10^4^ cells/ml) were prepared using Eagle's Minimum Essential Medium (non-serum) and transferred to the upper chamber with 0.1 ml per well. ATCC-formulated Eagle’s Minimum Essential Medium containing 20% fetal bovine serum was added into the bottome chamber of each well. Cells were cultured at 37 °C and 5% CO_2_ for 2 h and upper chamber membranes were stained for 20 min at room temperature with 0.5% crystal violet (Sigma-Aldrich). An optical microscope was used to count migrating and invading cells.

### Statistical analysis

Data were expressed as mean ± standard deviation (SD) of three replicates. Correlations of POU3F3 and MEG3 with AJCC stages were analyzed by Chi square test. Correlations between expression levels of POU3F3 and MEG3 were analyzed by Pearson’s correlation coefficient. Comparisons between paired tumor and normal tissues were performed by paired *t* test. Comparisons of multiple cell transfection groups were performed by ANOVA Tukey’s test. Differences with *p* < 0.05 were statistically significant.

## Results

### POU3F3 was upregulated in melanoma tissues and was correlated with AJCC stage

**Fig. 1:**
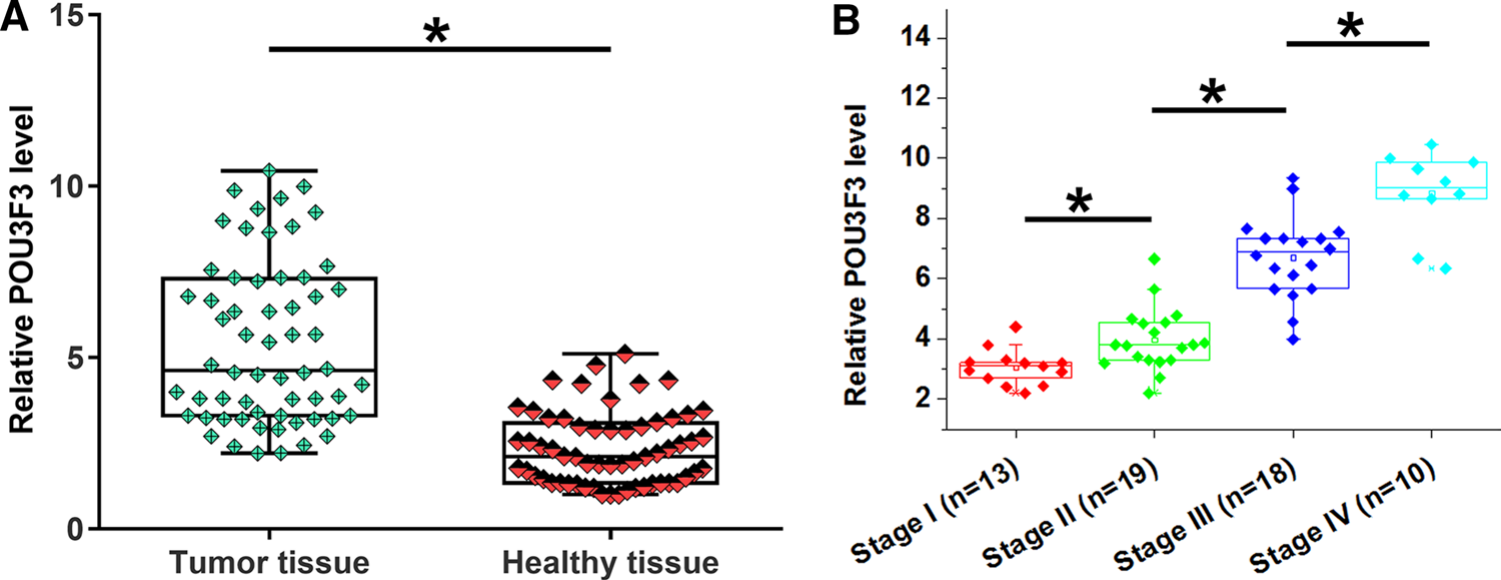
POU3F3 was upregulated in melanoma tissues compared with the normal tissues. Tumor and paired normal tissues from 60 melanoma patients were subjected to RNA isolation and RT-qPCR to analyze the differential expression of POU3F3 in melanoma. RT-qPCR data were compared by paired t test. Compared to normal tissues, POU3F3 expression levels were significantly increased in tumor tissues (**A**). Expression of POU3F3 in tumor tissues was analyzed among patients with different clinical stages, and POU3F3 expression levels in tumor tissues increased with the progression of cancer (**B**). * *p* < 0.05

POU3F3 expression in paired tumor and healthy tissues was detected by RT-qPCR. Compared to normal tissues, POU3F3 expression level was significantly higher in tumor tissues (Fig. 1A, *p* < 0.05). In addition, POU3F3 level in tumors increased with melanoma progressing from stage I to IV (Fig. 1B, p < 0.05). Based on Youden’s index, patients were divided into POU3F3 high and low expression groups. Correlations between POU3F3 expression and AJCC stages were analyzed by Chi square test. The results showed that POU3F3 level in tumor tissues was significantly correlated with AJCC stages (Chi square = 23.33, *p* < 0.01).

### MEG3 was downregulated in tumor tissues and inversely correlated with POU3F3

**Fig. 2:**
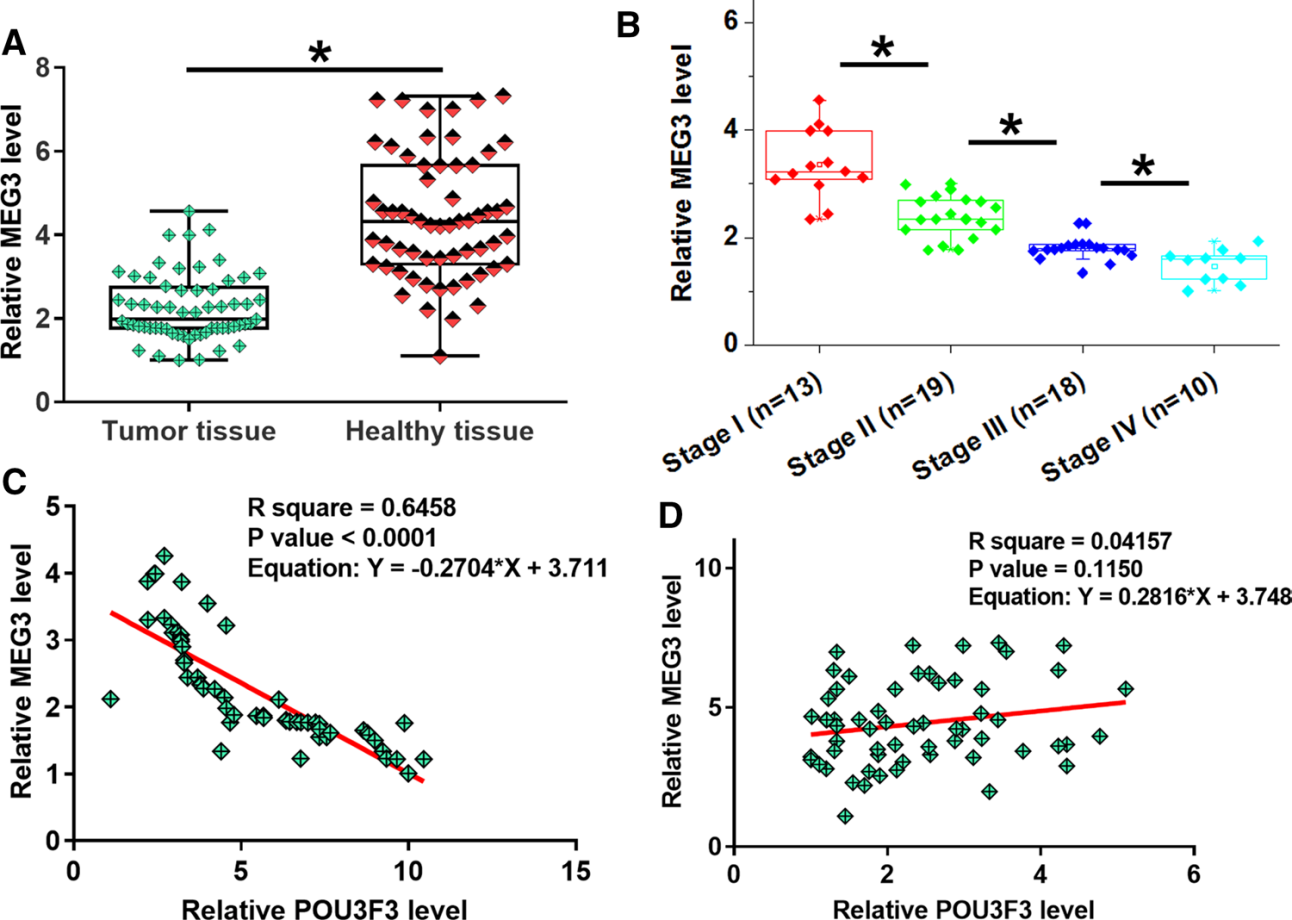
MEG3 was downregulated in tumor tissues and inversely correlated with POU3F3 in melanoma tissues. The differential expression of POU3F3 in melanoma was also analyzed by measuring its expression levels in paired tumor and normal tissue samples collected from 60 melanoma patients. RT-qPCR data were compared by paired t test. Compared with adjacent normal tissues, MEG3 expression levels were significantly decreased in tumor tissues (**A**).* *p* < 0.05. Expression of MEG3 in tumor tissues was analyzed among patients with different clinical stages, and MEG3 expression level in tumor tissues decreased with the progression of cancer (**B)**. Pearson’s correlation coefficient revealed that expression levels of POU3F3 and MEG3 were significantly and inversely correlated in tumor tissues (**C**) but not in normal tissues (**D**)

MEG3 expression in paired tissues was also detected by RT-qPCR. Compared to normal tissues, MEG3 expression level was significantly lower in tumor tissues (Fig. 2A, p < 0.05). Moreover, MEG3 levels in tumors decreased with melanoma progressing from stage I to IV (Fig. 2B, p < 0.05). Correlations between POU3F3 and MEG3 were analyzed by Pearson’s correlation coefficient. It was observed that POU3F3 and MEG3 were significantly and inversely correlated across tumor tissues (Fig. 2C) but not normal tissues (Fig. 2D). It is worth noting that MEG3 expression levels in tumor tissues were also significantly correlated with AJCC stages (Chi square = 17.61, *p* < 0.01).

### POU3F3 was an upstream inhibitor of MEG3 in melanoma cells

**Fig. 3:**
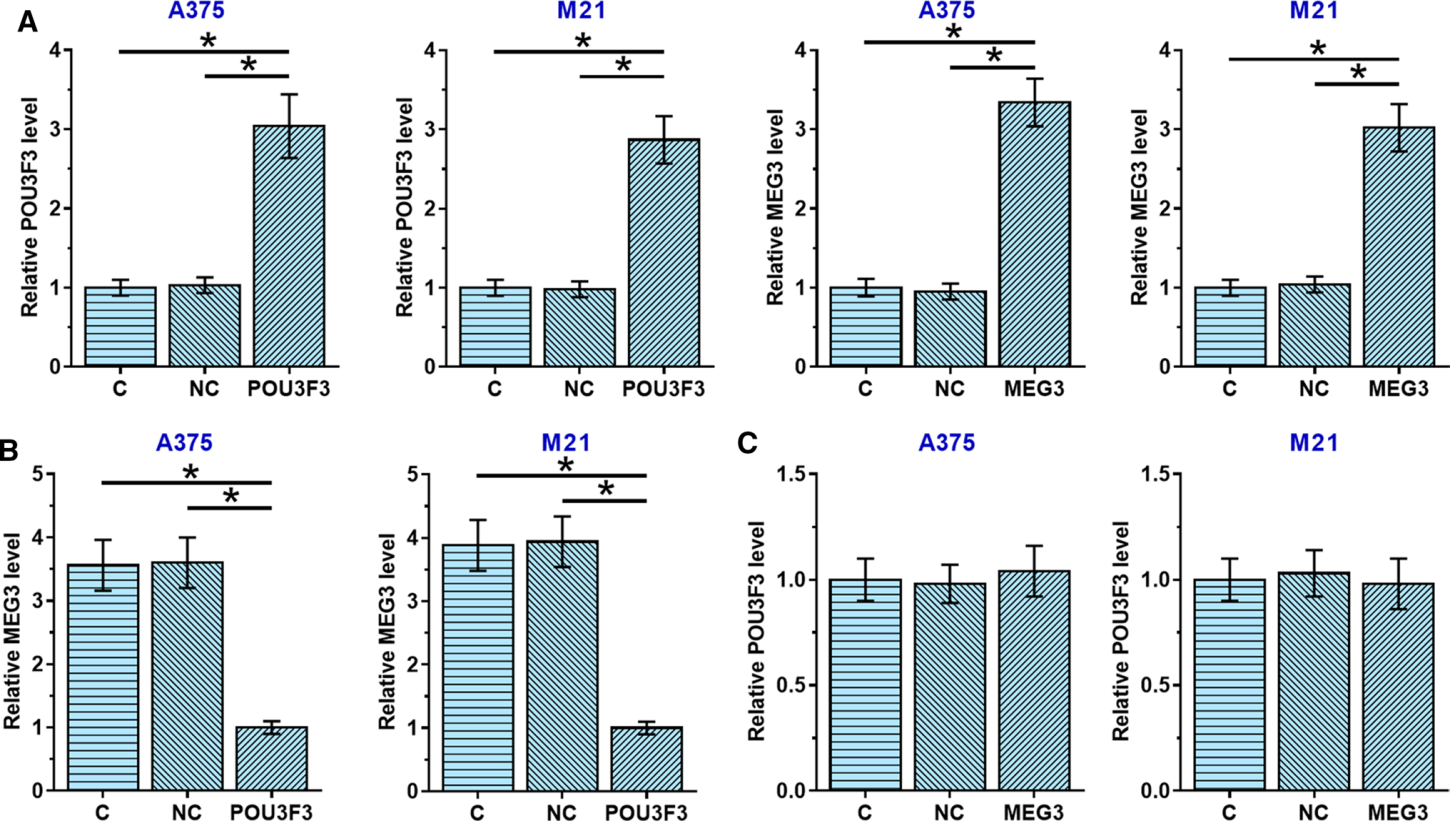
Overexpression experiments revealed the role of POU3F3 as an upstream inhibitor of MEG3 in melanoma cells. A375 and M21 cells were transfected with POU3F3 or MEG3 expression vector. RT-qPCR data analyzed by ANOVA (one-way) and Tukey test showed that POU3F3 and MEG3 expression levels were significantly increased in cells of A375 and M21, two human melanoma cell lines, at 24 h after transfections compared to control (C) and negative control (NC) groups (**A**). Compared to C and NC groups, POU3F3 overexpression led to MEG3 inhibition in melanoma cells (**B**), while MEG3 overexpression failed to affect POU3F3 (**C**). C, untransfected cells; NC, cells transfected with empty vector. *p < 0.05

POU3F3 and MEG3 were overexpressed in A375 and M21 cells, two human melanoma cell lines, to further investigate their interactions in melanoma. As shown in Fig. 3A, POU3F3 and MEG3 overexpression were achieved in both cell lines (*p* < 0.05). Expression levels of POU3F3 and MEG3 were normalized to the control cells. No significant differences in expression levels of POU3F3 (Fig. 3B) and MEG3 (Fig. 3C) were observed between control (C, untransfected cells) and negative control (NC, cells transfected with empty vector) groups (*p* > 0.05). However, compared to C and NC groups, POU3F3 overexpression decreased MEG3 expression in melanoma cells (Fig. 3B, p < 0.05), while MEG3 overexpression failed to significantly affect POU3F3 (Fig. 3C, p < 0.05).

### The interaction between MEG3 and POU3F3 participates in the regulation of proliferation but not migration and invasion of melanoma cells

**Fig. 4:**
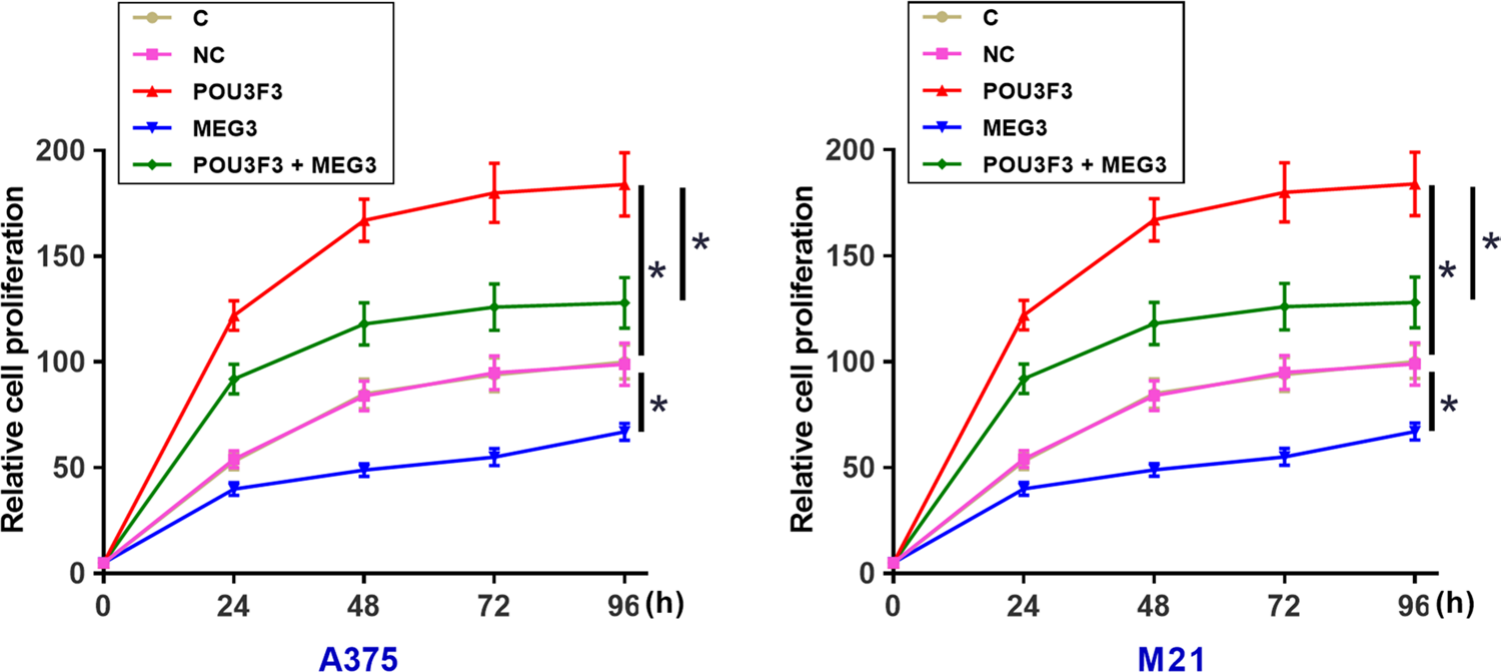
The interaction between POU3F3 and MEG3 participates in the regulation of proliferation of melanoma cells. A375 and M21 cells with POU3F3 and/or MEG3 overexpression were subjected to cell proliferation assay. Cell proliferation data analyzed by ANOVA (one-way) and Tukey test showed that, compared to C and NC groups, POU3F3 overexpression promoted, while MEG3 overexpression inhibited melanoma cell proliferation. In addition, the rescue experiment showed that MEG3 overexpression attenuated the enhancing effects of POU3F3 overexpression on cancer cell proliferation. C, untransfected cells; NC, cells transfected with empty vector. *p < 0.05

**Fig.5:**
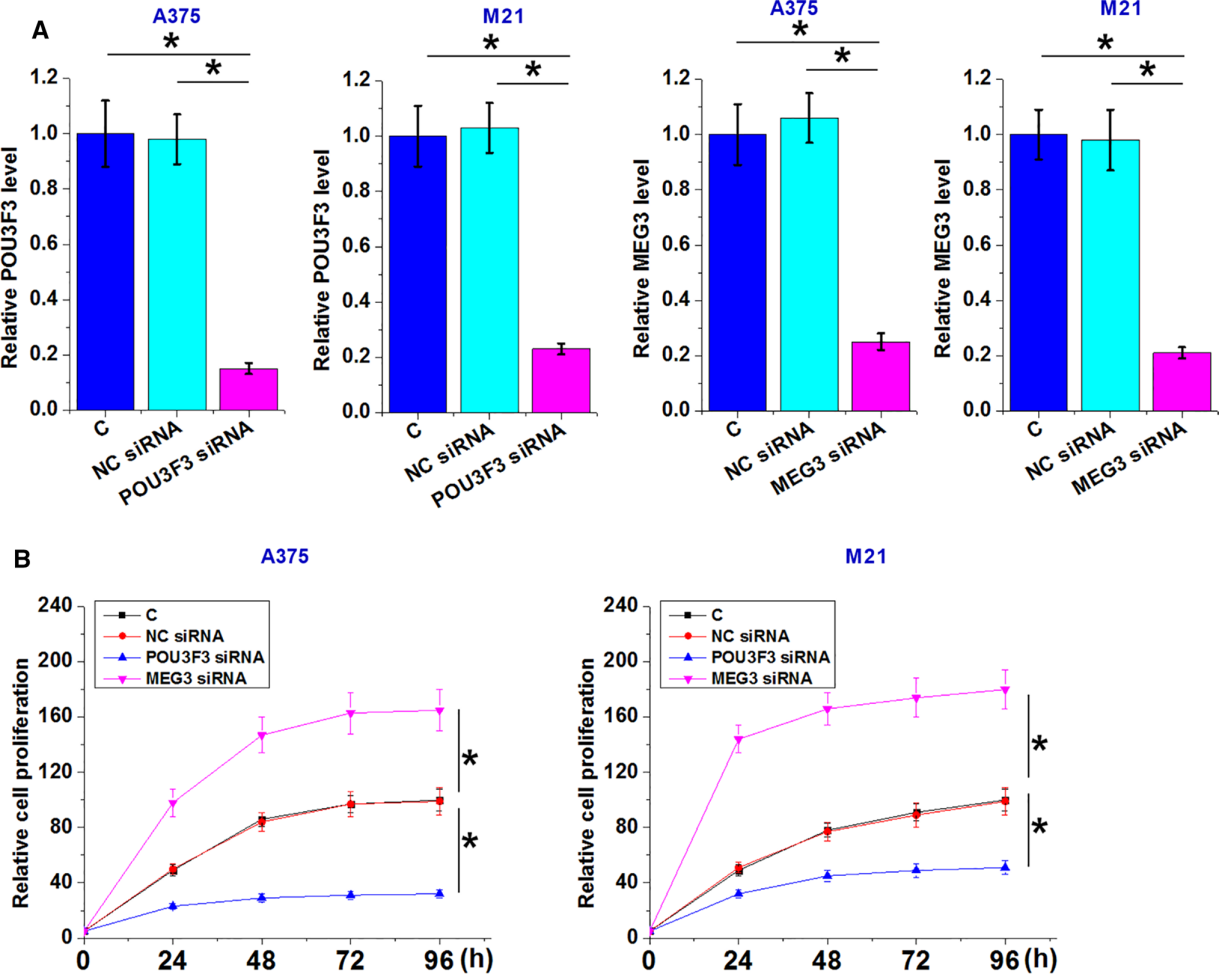
Analysis of the role of POU3F3 and MEG3 downregulation in the proliferation of A375 and M21 cells. POU3F3 and MEG3 downregulation was achieved by transfecting their siRNAs in both A375 and M21 cells (**A**). POU3F3 downregulation decreased cell proliferation and MEG3 downregulation increased cell proliferation (**B**). C, untransfected cells; NC miRNA, cells transfected with NC siRNA. * *p* < 0.05

CCK-8 assay was performed to measure cell proliferation abilities after transfections. Analysis of CCK-8 assay data showed that POU3F3 overexpression increased melanoma cell proliferation, while MEG3 overexpression decreased melanoma cell proliferation (Fig. 4, *p* < 0.05). In addition, the rescue experiment showed that MEG3 overexpression attenuated the enhancing effects of POU3F3 overexpression on cancer cell proliferation (Fig. 4, *p* < 0.05). However, POU3F3 overexpression failed to significantly affect cell invasion and migration (data not shown). POU3F3 and MEG3 downregulation was also achieved by transfection of their siRNA in both A375 and M21 cells (Fig. 5A, p < 0.05). CCK-8 assay showed that POU3F3 downregulation decreased cell proliferation while MEG3 downregulation increased cell proliferation (Fig. 5B, p < 0.05).

## Discussion

Our study revealed the oncogenic function of POU3F3 in melanoma. In addition, the interaction between POU3F3 and MEG3 characterized in this study provided new insights into the pathogenesis of melanoma.

The development and progression of cancer are multistep processes. Tumor growth provides the basis for all malignant cancer behaviors, such as tumor metastasis. However, studies on tumor microenvironment and genome-wide gene expression revealed that tumor growth and metastasis are two relatively independent processes that require the involvement of different genetic factors [19, 20]. POU3F3 has been characterized as an oncogenic lncRNA in many types of cancers [12–14]. In breast cancer, POU3F3 suppresses cancer cell apoptosis and induces cell proliferation by inactivating caspase 9 [12]. In nasopharyngeal carcinoma, POU3F3 activates TGF-β1 to promote cancer cell invasion and migration [13]. In prostate carcinoma, POU3F3 promotes cancer cell proliferation by upregulating ROCK1 [14]. In the present study, we reported that POU3F3 overexpression only significantly affected the proliferation but not the migration and invasion of melanoma, indicating the specific involvement of POU3F3 in the regulation of melanoma growth.

Functions of lncRNAs in cancer biology are mostly achieved through interactions with other signaling molecules [21]. It has been reported that lncRNAs may interact with other non-coding RNAs, such as microRNAs to achieve their biological functions [22–24]. However, studies of the interactions between different lncRNAs are rare. In the present study, we proved that POU3F3 plays a role as the upstream inhibitor of MEG3 in regulating melanoma cell proliferation. Our study provided new insights into the pathogenesis of melanoma. It is worth noting that MEG3 is a well-studied tumor suppressive lncRNA. For instance, MEG3 can upregulate p53 to inhibit pancreatic cancer cell proliferation [16]. To our best knowledge, POU3F3 and MEG3 do not encode any protein and are true non-coding RNAs. Therefore, we observed the interaction between these two lncRNAs.

Interestingly, a recent study reported that MEG3 inhibited the migration and invasion of melanoma through its interactions with Wnt signaling pathway [17]. In the present study, POU3F3 overexpression downregulated MEG3 but failed to significantly affect the migration and invasion of melanoma cells. Therefore, POU3F3 could also interact with other downstream pathways to reverse the enhancing effects of MEG3 inhibition on cancer cell migration and invasion. This hypothesis is supported by the following observations: (1) MEG3 overexpression only partially rescued the promoted cell proliferation caused by POU3F3 overexpression, indicating the interaction between POU3F3 and other factors; and (2) expression levels of POU3F3 and MEG3 were significantly and inversely correlated in tumor tissues but not in normal tissues, indicating the existence of pathological mediators between POU3F3 and MEG3.

## Conclusion

In conclusion, POU3F3 is upregulated in melanoma and POU3F3 overexpression may promote melanoma cell proliferation by downregulating MEG3.

## Data Availability

The datasets used and/or analyzed during the current study are available from the corresponding author on reasonable request.

## Abbreviations

POU3F3: LncRNA POU3F3
MEG3: LncRNA MEG3
RT-qPCR: Real-time quantitative PCR
CCK-8: Cell counting kit-8
